# Effect of Metformin (MTF) Intervention During Pregnancy in Women With Polycystic Ovarian Syndrome (PCOS): A Systematic Review

**DOI:** 10.7759/cureus.44166

**Published:** 2023-08-26

**Authors:** Srishti Kanda, Uzair Chatha, Victor A Odoma, Aakanksha Pitliya, Esraa M AlEdani, Japneet K Bhangu, Khalid Javed, Prabhleen Kaur Manshahia, Shamsun Nahar, Pousette Hamid

**Affiliations:** 1 Internal Medicine, California Institute of Behavioral Neurosciences & Psychology, Fairfield, USA; 2 Medicine, Lahore Medical and Dental College, Lahore, PAK; 3 Cardiology/Oncology, Indiana University (IU) Health, Bloomington, USA; 4 Anesthesiology, Internal Medicine, California Institute of Behavioral Neurosciences & Psychology, Fairfield, USA; 5 Medicine, All India Institute of Medical Sciences, Rishikesh, IND; 6 Neurology, California Institute of Behavioral Neurosciences & Psychology, Fairfield, USA

**Keywords:** pregnancy-induced hypertension (pih), fetal macrosomia, live birth rate, preterm delivery, body mass index: bmi, miscarriage, clinical pregnancy rate, : pregnancy, polycystic ovarian disease, “ metformin” “antidiabetic drugs”

## Abstract

Metformin (MTF) is a commonly prescribed medication for women with polycystic ovarian syndrome (PCOS), but its impact on pregnancy outcomes in women with PCOS remains controversial. This systematic review aims to evaluate the effects of MTF intervention on pregnancy outcomes in women with PCOS and the impact of MTF on offspring. A comprehensive search is conducted in PubMed, Google Scholar, and ScienceDirect databases from 2019 up to May 16, 2023. Only review articles and meta-analyses are included, focusing on women with PCOS who received MTF during pregnancy or as part of infertility treatment. The primary outcomes of interest are clinical pregnancy rate (CPR), miscarriage rate, preterm birth rate, and live birth rate. Secondary outcomes are the safety profile of MTF. Data extraction and quality assessment are performed following the Preferred Reporting Items for Systematic Reviews and Meta-Analyses (PRISMA) guidelines and the assessment using the multiple systematic reviews (AMSTAR) 2 tool, respectively. The initial search produced 1877 studies. Thirteen studies were included in the review. While the use of MTF during pregnancy in women with PCOS may have some benefits in reducing certain pregnancy complications such as pregnancy-induced hypertension (PIH), gestational diabetes mellitus (GDM), preeclampsia, preterm delivery, reducing the risk of ovarian hyperstimulation syndrome (OHSS) in women with PCOS undergoing in vitro fertilization (IVF); however, there is no significant difference in clinical pregnancy and live birth rates overall, but subgroup analysis suggests potential benefits for women with a higher body mass index (BMI). MTF is associated with a larger fetal head circumference and potential long-term effects on offspring's BMI and obesity. Further research is needed to better understand the optimal dosing of MTF, long-term effects, and effects in specific subgroups. The heterogeneity of the included studies limited the ability to analyze the data effectively, leading to challenges in drawing definitive conclusions.

## Introduction and background

Polycystic ovarian syndrome (PCOS) is a complex disease characterized by elevated androgen levels, metabolic abnormalities, and disrupted ovulation [1]. PCOS stands as the primary cause of infertility, affecting approximately 8%-13% of women in their reproductive years. Studies indicate that the combination of androgen excess, insulin resistance, and hypothalamic-pituitary dysfunction significantly contributes to ovarian irregularities, thereby leading to anovulation and infertility [2].

**Figure 1:**
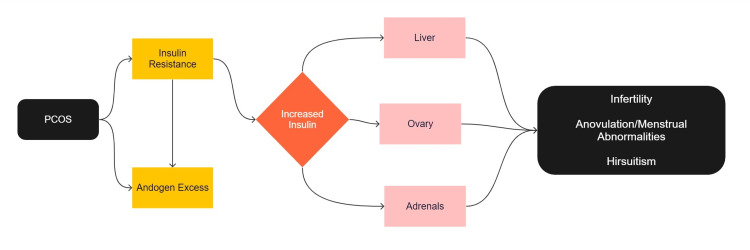
Pathophysiology of polycystic ovarian syndrome (PCOS) PCOS: Polycystic ovarian syndrome This figure was created by the first author Srishti Kanda.

Insulin resistance (IR) has been identified as a major factor in PCOS-related infertility. Elevated insulin levels are often present in women with PCOS due to IR. IR is exacerbated by hyperandrogenic features and obesity and is significantly increased during pregnancy in women with PCOS [3]. This can lead to difficulties in embryo implantation and adverse pregnancy outcomes such as miscarriage and preterm birth. This is particularly distressing for women who have difficulty conceiving. Figure 1 shows the pathophysiology of PCOS.

**Figure 2:**
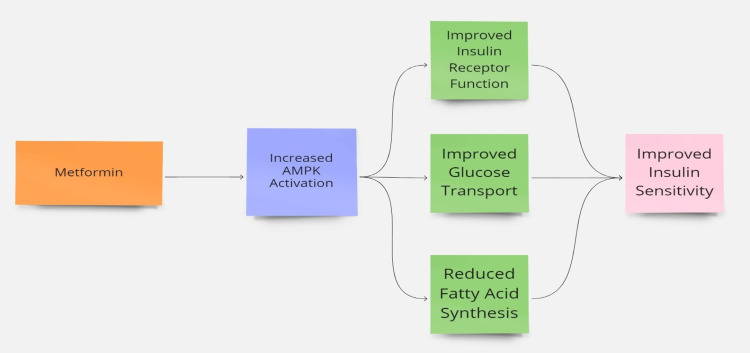
Mechanism of metformin in insulin sensitivity AMPK: Adenosine monophosphate-activated protein kinase The figure was created by the first author Srishti Kanda.

Various treatment strategies exist for PCOS. Metformin (MTF), a well-known hypoglycemic medication, has emerged as a potential drug due to its remarkable ability to enhance ovulation, increase insulin sensitivity, and promote clinical pregnancies in women with PCOS [4,5]. MTF reduces gluconeogenesis, inhibits lipid synthesis, and decreases glucose absorption in the gastrointestinal tract. While MTF has been used for many years to induce ovulation in women with PCOS, it is not recommended as a first-line treatment [6]. Many women conceive while using MTF; therefore, it is essential to ensure that it is safe to take during early pregnancy. Multiple clinical trials and meta-analyses (MA) have been conducted to assess the efficacy of MTF in enhancing pregnancy outcomes for infertile women with PCOS. However, the potential benefits and risks of MTF remain unclear [7]. This systematic review (SR) aims to evaluate the current literature on the effects of MTF intervention during pregnancy in women with PCOS with an aim to address key questions regarding effectiveness, clinical pregnancy outcomes (CPO) including miscarriages, preterm and live births, and safety of MTF treatment during pregnancy in women with PCOS. Figure 2 depicts the mechanism by which MTF increases insulin sensitivity. 

## Review

Methods

This SR was done using the Preferred Reporting Items for Systematic Reviews and Meta-Analysis (PRISMA) guideline [8].

Inclusion Criteria

Primary research was performed using PubMed, Google Scholar, and ScienceDirect. Articles over the past five years on the impact of MTF intervention in pregnancy in women with PCOS have been selected. Articles were selected if they also met the criteria for free full text in English and were conducted between 2019 and May 16, 2023. Using the Medical Subject Headings (MeSH) on-demand strategy, the keywords “metformin,” “polycystic ovarian syndrome,” “pregnancy outcome,” and “female” were used.

Exclusion Criteria

We excluded studies conducted before 2019, languages other than English, studies involving animals, paid articles, gray literature, and wrong intervention.

Search Strategy

**Figure 3:**
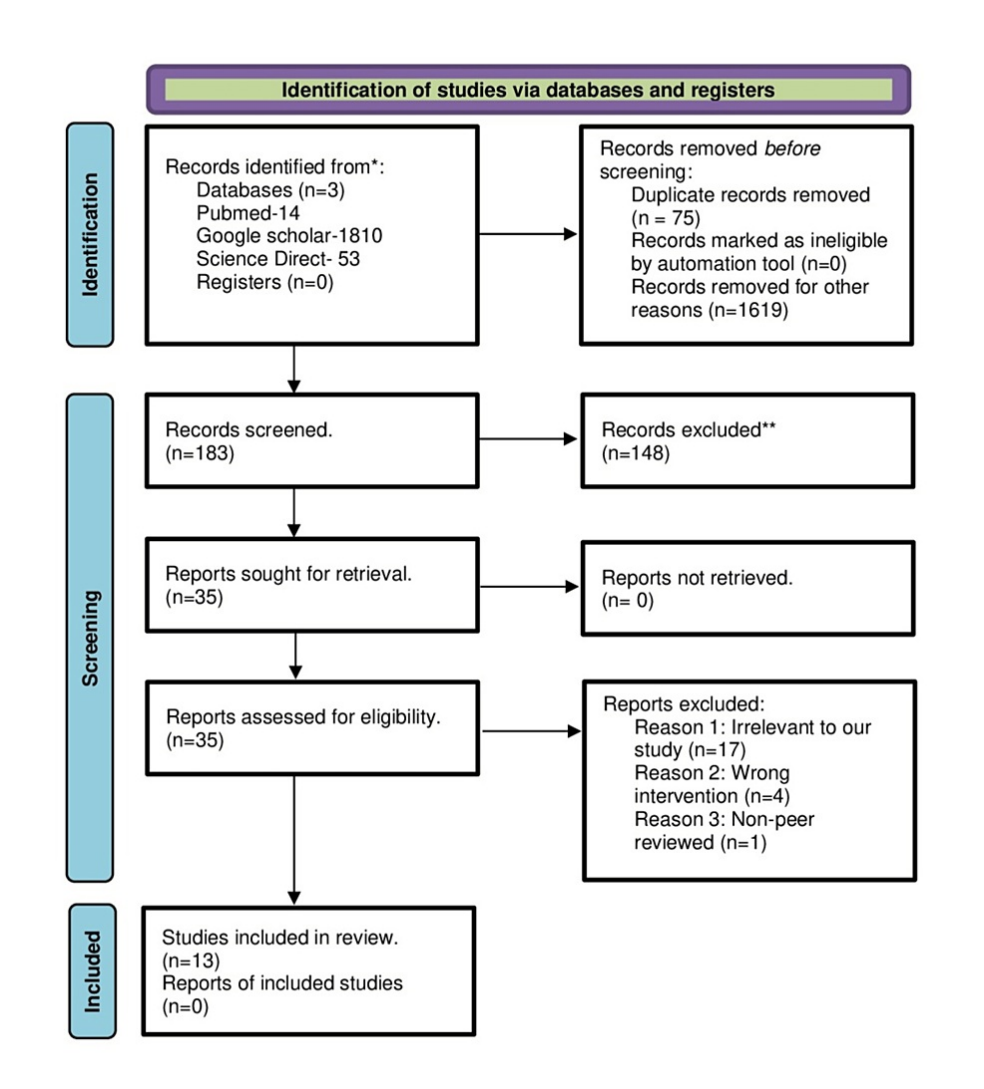
PRISMA flowchart demonstrating the selection process of the included articles PRISMA: Preferred reporting items for systematic reviews and meta-analyses

PubMed was explored using MeSH on demand or search items as follows ("Pregnancy"[MeSH Terms] OR "Gestation" OR "Pregnancy") AND ("Female"[MeSH Terms] OR "Female" OR "Females") AND ("Metformin (MTF)"[MeSH Terms] OR "Dimethylbiguanidine" OR "Dimethylguanylguanidine" OR "Glucophage" OR "Metformin (MTF)" OR "Metformin (MTF) HCl" OR "Metformin (MTF) Hydrochloride") AND ("Polycystic Ovary Syndrome"[MeSH Terms] OR "Polycystic Ovarian Syndrome" OR "Polycystic Ovary Syndrome" OR "Polycystic Ovary Syndrome 1" OR "Sclerocystic Ovarian Degeneration" OR "Sclerocystic Ovaries" OR "Sclerocystic Ovary Syndrome" OR "Stein-Leventhal Syndrome. ScienceDirect was searched for research articles with open access in the last five years (2019-2023) using the keywords “Metformin,” “PCOS,” and “Pregnancy.” Google Scholar was searched for review articles in the last five years (2019-2023) using keywords “PCOS,” “Metformin,” and “Pregnancy outcome.” A total of 1,877 articles were included, and 35 articles were evaluated for eligibility and quality after removing duplicates and irrelevant articles. Two reviewers scanned the titles and abstracts independently. A third reviewer intervened to resolve conflicts. Articles that were irrelevant, with the wrong intervention, and non-peer reviewed were removed. Finally, we included 13 articles in our study, as presented in the PRISMA flow diagram in Figure 3. 

Study Quality Appraisal

Each article was analyzed separately for quality appraisal and the potential risk of bias. Meta-analyses and SRs were evaluated using the Assessment of Multiple Systematic Reviews 2 (AMSTAR 2) tool [9]. The review articles were subjected to the Scale for the Assessment of Narrative Review Articles (SANRA) [10]. Articles were scored as either high quality, low quality, or unclear.

Results

The SR identified 13 relevant articles that analyzed the effects of MTF intervention during pregnancy in women with PCOS. Table 1 summarizes the key findings of each study [11-24].

**Table 1:** Results of each study included in the review IVF: in vitro fertilization; ICSI: intracytoplasmic sperm injection; PCOS: polycystic ovarian syndrome; MTF: metformin; RCT: randomized controlled trial; OHSS: ovarian hyperstimulation syndrome; CPR: clinical pregnancy rate; GnRH: gonadotropin-releasing hormone; BMI: body mass index; GDM: gestational diabetes mellitus; PGDM: pregestational diabetes mellitus; MCM: major congenital malformations; ICSI-ET: intracytoplasmic sperm injection and embryo transfer; EMT: endometrial thickness; ER: endometrial receptivity; MR: miscarriage rate; PIH: pregnancy-induced hypertension; CC: clomiphene citrate.

Article	Author	Study design	Participants	Conclusion
Metformin treatment before and during IVF or ICSI in women with polycystic ovary syndrome [11]	Tso et al. [11] (2020)	Systematic review	Women of reproductive age with anovulation attributed to PCOS, with or without other cause of infertility, who were treated with metformin (MTF) before and during an in vitro fertilization (IVF) or intracytoplasmic sperm injection (ICSI) cycle	In comparison to placebo or no treatment, MTF had an undetermined impact on the live birth rate per woman. Low-quality evidence indicates that metformin may lower the live birth rate when compared to placebo or no therapy. Eleven randomized controlled trials (RCTs) with 1,091 women found that MTF may lower the risk of ovarian hyperstimulation syndrome (OHSS). MTF may increase clinical pregnancy rate (CPR) per woman as compared to placebo/no therapy when using a lengthy protocol gonadotropin-releasing hormone (GnRH) agonist. MTF's impact on CPR for each woman using a short protocol GnRH antagonist was unknown when compared to placebo or no therapy. The live birth rate did not differ significantly between women who got MTF and those who did not. There was an increase in CPR in women with body mass index (BMI) greater than or equal to 26 receiving MTF therapy.
Generational health impact of PCOS on women and their children [12]	Hart [12] (2019)	Review article	PCOS women	Maternal exposure to MTF during the first trimester of pregnancy showed no increase in fetal malformations. Children exposed to MTF in utero during pregnancy were found to be heavier in childhood.
Improvement effect of metformin on female and male reproduction in endocrine pathologies and its mechanisms [13]	Shpakov [13] 2021)	Review article	Women with diagnosed PCOS	Absence of teratogenic effect of MTF and the low risks of MTF therapy on the health of the mother and child.
Current therapeutic use of metformin during pregnancy: maternal changes, postnatal effects, and safety [14]	Quadir [14] (2021)	Review article		No evidence of increased risk for miscarriages or congenital malformations. Use of MTF during pregnancy shows a significant reduction in preeclampsia and gestational diabetes mellitus (GDM).
Major malformations risk following early pregnancy exposure to metformin: a systematic review and meta-analysis [15]	Abolhassani et al. [15] (2023)	Systematic review and meta-analysis	Metformin exposure during the first trimester in women with PCOS and pre-gestational diabetes mellitus (PGDM)	The use of metformin during the first trimester of pregnancy in women with PCOS or PGDM does not significantly increase the overall risk of MCM.
Association of metformin with pregnancy outcomes in women with polycystic ovarian syndrome undergoing in vitro fertilization: a systematic review and meta-analysis [16]	Wu et al. [16] (2020)	Systematic review and meta-analysis	Twelve RCTs with 1,123 women having PCOS were treated for infertility with in vitro fertilization/ intracytoplasmic sperm injection and embryo transfer (IVF/ICSI-ET)	The risk of OHSS in women randomized to MTF was lower than in women not randomized to MTF, although this difference was not significant for women with PCOS with a BMI below 26. In a post hoc analysis, treatment with MTF was associated with an increased clinical pregnancy rate in women with a BMI of 26 or higher.
Metformin during pregnancy: effects on offspring development and metabolic function [17]	Jorquera et al. [17] (2020)	Review article	Metformin (MTF) use during pregnancy in women with PCOS and type 2 diabetes mellitus	Metformin reduces the development of GDM in pregnant women with PCOS.
The function of metformin in endometrial receptivity (ER) of patients with polycyclic ovary syndrome (PCOS): a systematic review and meta-analysis [18]	Yuan et al. [18] (2021)	Systematic review and meta-analysis	Sixty-two eligible studies including 6,571 PCOS patients	Metformin improved CPR and decreased miscarriage (MR) in PCOS patients because of increased endometrial thickness (ET) and decreased endometrial artery resistance index.
Effects of metformin on pregnancy outcome, metabolic profile, and sex hormone levels in women with polycystic ovary syndrome and their offspring: a systematic review and meta-analysis [19]	Zhu et al. [19] (2022)	Systematic review and meta-analysis	Women diagnosed with PCOS	Metformin treatment significantly reduced the risk of preterm delivery, pregnancy-induced hypertension (PIH), preeclampsia, and fetal macrosomia. In fetuses, MTF was significantly associated with larger head circumference and higher long-term body mass index (BMI).
Gestational metformin administration in women with polycystic ovary syndrome: a systematic review and meta-analysis of RCT studies [20]	Cao et al. [20] (2021)	Systematic review and meta-analysis	Six RCT studies involving 1,229 participants were included	Metformin use was associated with reduced risk of preterm delivery and larger neonatal head circumference but had no effect on the incidence of GDM, miscarriage, preeclampsia, and neonatal length and birth weight.
Management strategy of infertility in polycystic ovary syndrome [21]	Li et al. [21] (2022)	Review article	Women with diagnosed PCOS and anovulation	Studies comparing metformin with clomiphene citrate (CC) have shown that the pregnancy rate and live birth rate with MTF alone are significantly reduced. The chance of conception with CC therapy is significantly increased (approximately twice). For patients with CC resistance, combining metformin with CC may increase the pregnancy rate, but it has not shown an increase in the live birth rate. MTF alone can increase ovulation but has limited benefits in improving live birth rates.
A review: Brief insight into polycystic ovarian syndrome [22,23].	Bulsara et al. [23] (2021)	Review article	Women with diagnosed PCOS	The use of MTF in pregnancy does not show any teratogenic effect. MTF reduces inflammation and complications related to pregnancy.
Does metformin improve reproduction outcomes for nonobese, infertile women with polycystic ovary syndrome? Meta-analysis and systematic review [24]	Magzoub et al. [24] (2022)	Meta-analysis and systematic review	Eleven RCTs, including 2,638 patients with PCOS	In nonobese women with PCOS, MTF is associated with a slight increase in CPR compared to placebo. MTF is comparable to CC when the outcome is CPR and the risk of multiple pregnancies tends to be lower. MTF had a higher risk of miscarriage rate. Adding MTF to CC treatment decreases miscarriage risk by twofold compared to MTF alone and showed no difference compared to CC alone.

Discussion

The current SR aims to assess the impact of MTF treatment during pregnancy in women diagnosed with PCOS by analyzing data from multiple sources. The combined evidence provided valuable insights into the outcomes of MTF intervention compared to receiving no treatment/alternate treatment or placebo across various aspects of pregnancy outcomes.

Live Birth Rate

In terms of live birth rate per woman, the results indicated uncertainty regarding the effect of MTF compared to no treatment or placebo [11]. It should be noted that the quality of the evidence supporting this conclusion was assessed as low. One study using a short regimen of GnRH antagonists reported lower survival with MTF than with no treatment or placebo (RR 0.48). This implies that the chance of survival with a placebo is higher than with MTF [11]. However, the evidence supporting this result is also of low quality. Another study that compared MTF with clomiphene citrate (CC) found a significant reduction in pregnancy and live birth rates with MTF alone [21]. For CC-resistant patients, the combination of MTF with CC may increase pregnancy rates, but not live births [21]. 

Clinical Pregnancy Rate (CPR) and Miscarriage Rate (MR)

With long-term GnRH agonist stimulation, MTF is likely to increase the CPR per woman compared with placebo. However, with short-term GnRH antagonist regimens, the effect of MTF on clinical pregnancy rates remains uncertain [11]. The evidence supporting these results is of low quality and very low quality, respectively. When combined with clomiphene citrate (CC), MTF has been shown to increase ovulation and pregnancy rates in infertile women with PCOS [25]. One study showed that MTF improves CPR and reduces MR in PCOS patients due to increased endometrial thickness (EMT) and decreased endometrial artery resistance index [18]. Another study also showed a slight increase in CPR with MTF use in nonobese women with PCOS compared with placebo, but this resulted in a higher risk of miscarriage rate with MTF only. Adding MTF to CC treatment resulted in a reduction in MR compared with MTF alone and did not find a difference if treated with CC only [24].

Regarding the rate of miscarriage per woman, data suggest uncertainty regarding the effects of MTF compared with no treatment or placebo [11]. The evidence supporting this finding was found to be of low quality. In terms of overall CPR and live births, the review found no significant differences between women who received MTF and those who did not [16]. However, a post hoc analysis revealed that in women with a BMI of 26 or higher, treatment with MTF was associated with an increased CPR. This suggests that MTF may have a positive effect on pregnancy outcome (PO) in women with a higher BMI [16].

Ovarian Hyperstimulation Syndrome (OHSS)

With regard to the occurrence of OHSS, there is pooled evidence that MTF may reduce morbidity compared with no treatment or placebo [11]. However, this was not statistically significant for PCOS women with a BMI below 26. This suggests that MTF may be particularly beneficial for women with a high BMI [16]. The quality of evidence for this outcome was found to be low. 

Impact of MTF on Pregnancy Complications

It was found that MTF treatment during pregnancy reduced the risk of PIH, preeclampsia, macrosomia (abnormally large birth weight), and preterm delivery compared to placebo [19,14,20]. These findings are significant considering that women with PCOS are at an increased risk of developing complications such as gestational diabetes mellitus (GDM), PIH, and preterm delivery [26,17]. The findings of this study are consistent with a number of high-quality studies supporting the benefits of MTF in reducing the risk of pregnancy complications [19].

Impact on Offspring

Regarding the impact on offspring, the review indicated that MTF treatment during pregnancy decreased the risk of macrosomia (large birth weight) but was associated with larger fetal head circumference [19]. There was no evidence of an association between MTF treatment and fetal birth weight or length. Long-term follow-up studies showed that children exposed to MTF during pregnancy tended to have a higher mean BMI and an increased prevalence of obesity in childhood [19,12]. These findings suggest that MTF may have a significant impact on the long-term growth and risk of obesity in offspring. In one study, major congenital malformation (MCM) rates in randomized control trials (RCTs) and observational studies were OR 0.93 and OR 1.35, respectively, indicating no increased risk of MCM rates with first-trimester MTF exposure [15]. The review also addressed the safety of MTF use during pregnancy. It concluded that MTF does not show any teratogenic effects and can reduce inflammation and complications related to pregnancy [22,13,23].

Limitations

The inclusion of only review articles and meta-analyses from specific databases (PubMed, Google Scholar, and Science Direct), published in the last five years in English with free full texts leads to a possibility of missing relevant research studies that may provide different perspectives on the topic. While the review briefly mentioned long-term effects on offspring, such as increased BMI and obesity risk, the extent and significance of these effects were not extensively explored.

## Conclusions

The systematic review provides evidence supporting the use of MTF treatment in pregnant women with PCOS. It highlights the potential benefits of MTF in reducing the risk of OHSS and pregnancy complications such as PIH, GDM, macrosomia, and preterm birth. It may improve CPR in women with a higher BMI. However, there are potential risks associated with MTF, including increased fetal head circumference and a potential long-term impact on an offspring's BMI and risk of obesity.

There is a need for further research to better understand the optimal dosing schedule, long-term implications, and the effects of MTF on pregnancy outcomes, particularly in obese women with PCOS. Larger multicenter studies are necessary to provide more definitive conclusions and address unresolved questions. Nonetheless, the review supports the use of MTF during pregnancy in women with PCOS, considering its potential benefits in reducing complications and improving pregnancy outcomes. The potential risks and benefits of MTF use during pregnancy in PCOS women should be weighed on a case-by-case basis considering individual patient characteristics.
